# Effect of Preventive Analgesia with Nalbuphine and Dexmedetomidine in Endoscopic Sinus Surgery

**DOI:** 10.1155/2022/2344733

**Published:** 2022-05-31

**Authors:** Pan Yu, Jie Zhang, Yan Zou, Jun Wang

**Affiliations:** ^1^Department of Anesthesiology, Department of Pain, The Affiliated Huaian Hospital of Xuzhou Medical University, Huaian Second People's Hospital, Huaian, Jiangsu, China; ^2^Department of General Surgery, The Affiliated Huaian Hospital of Xuzhou Medical University, Huaian Second People's Hospital, Huaian, Jiangsu, China

## Abstract

**Background:**

The study was to assess the efficacy and safety of nalbuphine combined with dexmedetomidine for preventive analgesia in endoscopic sinus surgery.

**Methods:**

110 patients with deviation of the nasal septum were randomized into the nalbuphine group (group N), dexmedetomidine combined with nalbuphine group (group DN), and saline group (group C). Fifteen minutes before the induction of anesthesia, patients in group N were injected nalbuphine 0.2 mg/kg intravenously; patients in group DN received intravenous infusion of dexmedetomidine 0.5 *μ*g/kg and injection of nalbuphine 0.2 mg/kg; patients in group C received 0.9% saline. Mean arterial pressure (MAP), heart rate (HR), numerical rating scale (NRS) scores, quality of recovery-40 (QoR-40) scores, the need for remedial analgesia, the consumption of remifentanil and propofol, and the incidence of adverse reactions were recorded.

**Results:**

MAP, HR, and NRS scores of the DN group were significantly lower and the QoR-40 scores were higher than those of groups N and C (*P* < 0.001). The need for remedial analgesia, the consumption of remifentanil and propofol, and the incidence of nausea in the DN group were the lowest among the three groups (*P* < 0.001).

**Conclusion:**

Preventive analgesia with nalbuphine and dexmedetomidine in endoscopic sinus surgery can not only maintain hemodynamic stability but also reduce intraoperative anesthetic dosage, postoperative pain, and improve the quality of postoperative recovery without affecting the revival and extubation time.

## 1. Introduction

Endoscopic sinus surgery is increasing in the treatment of rhinosinusitis (CRS) and nasal polyposis because of its lower blood loss, rapid recovery, and lower incidence of postoperative complications. However, studies have found that moderate and severe pain may occur within 24 hours after endoscopic sinus surgery, with an incidence of 42.0–79.0%, increasing the incidence of complications such as lung disease, poor wound healing, and insomnia, which are not conducive to early postoperative recovery [1]. Therefore, it is very important to do a good job in analgesic management. Because of the narrow space and rich internal blood flow in the nasal cavity, hemodynamics are often required to be stable to provide a relatively clean surgical field.

Preventive analgesia is an intervention provided before the beginning of pain stimulation, which can reduce or prevent subsequent pain. By blocking the transmission of noxious stimulation to the central nervous system, preventive analgesia aims to reduce postoperative pain [2]. Dexmedetomidine, an alpha-2 agonist with sedative and analgesic effects, can maintain stable hemodynamics and almost no respiratory depression occurrence [3]. Studies have shown that dexmedetomidine can reduce the usage of opioids and postoperative pain [4]. Thus, dexmedetomidine has become an important part of multimodal analgesia [5]. Nalbuphine, a kappa-opioid receptor agonist and a partial mu-opioid receptor antagonist with analgesic effects similar to morphine, is widely used in moderate to severe pain [6]. Studies have confirmed the effect of dexmedetomidine in endoscopic sinus surgery, but there are few articles regarding the use of nalbuphine combined with dexmedetomidine for preventive analgesia in endoscopic sinus surgery. Its effectiveness and safety are uncertain; therefore, the purpose of this study was to assess the effect of nalbuphine combined with dexmedetomidine on hemodynamics and postoperative pain in endoscopic sinus surgery. We hypothesize that the combination of nalbuphine and dexmedetomidine for preventive analgesia can maintain stable hemodynamics during endoscopic sinus surgery, reduce postoperative pain, and improve the quality of postoperative recovery.

## 2. Materials and Methods

This study was authorized by the Ethical Committee of Affiliated Huai'an Hospital of Xuzhou Medical University (Chairperson Xu Jifan, protocol number: HEYLL202006), Huai'an, China, on 8 December 2020. It has been enrolled in the Chinese Clinical Trial Registry (https://www.chictr.org. cn; trial registration no. ChiCTR2100042231) on 16 January 2021, and the patients and their families have signed informed consent.

### 2.1. Patient Selection

110 patients aged 18–65 years with ASA physical status I or II with deviation of the nasal septum were selected for the study between February 2021 and September 2021. Patients with the following conditions were excluded from this study: severe hypertension, long-term use of analgesic drugs or cortisol drugs, bradycardia, Adams–Stokes syndrome, preexcitation syndrome, severe heart, kidney, or liver disease, endocrine metabolism or nervous system diseases, severe atrioventricular block, and history of alcohol addiction.

### 2.2. Study Design and Data Collection

Patients were randomly divided into three groups by a computer-generated random number table: the nalbuphine group (group N), dexmedetomidine combined with nalbuphine group (group DN), and normal saline group (group C). The randomized group number was kept in a sealed opaque envelope, and the drugs were prepared by nurse anesthetists. None of the doctors and nurses involved in anesthesia management knew the allocation. The data were collected by an anesthesiologist who was blinded to group allocation.

After entering the operating room, venous access was established routinely. Blood pressure (BP), heart rate (HR), electrocardiography, and oxygen saturation (SpO2) were monitored, and then, patients accepted mask oxygen inhalation before intubation at a rate of 3 L/min for at least 3 minutes. Before anesthesia induction, group N received intravenous injection of nalbuphine 2 mg/kg (Yichang Humanwell Pharma, Hubei, China); in group DN, 0.5 *μ*g/kg dexmedetomidine was infused intravenously for 15 minutes, and 0.2 mg/kg nalbuphine was injected intravenously; group C received the same volumes of normal saline at the same time. Anesthesia was induced with intravenous midazolam 0.05 mg/kg (1 mg/ml; Nhwa Pharma, Jiangsu, China), propofol 1.5 mg/kg (Guorui Medicine, Sichuan, China), sufentanil 0.5 *μ*g/kg (5 *μ*g/ml; Yichang Humanwell Pharma, Hubei, China), and rocuronium 0.6 mg/kg (Xianju Medicine, Zhejiang, China). Then, an endotracheal tube was inserted with inner diameters of 7.0 and 7.5 mm for women and men. Mechanical ventilation was maintained with a tidal volume of 6–8 ml/kg, and the end-tidal carbon dioxide partial pressure was kept at 35–40 mmHg by adjusting the respiratory rate and tidal volume. The appropriate depth of anesthesia was maintained by adjusting the infusion rate of propofol (4–10 mg/kg/h) and remifentanil (0.2*–*0.4 *μ*g/kg/min). Rocuronium was injected intermittently to maintain muscle relaxation, and the mean arterial pressure was controlled within 20% of the baseline values. When the patient regained consciousness and spontaneous breathing, the tracheal tube was removed. After 15 minutes of observation, they were transferred to the ward. Tramadol 50 mg was injected intravenously if patients reported pain ≥4 on the numerical rating scale (NRS), and azasetron 10 mg was given when nausea and vomiting occurred.

The primary outcomes were HR and MAP before anesthesia (T1), at the time of intubation (T2), at the beginning of the operation (T3), at the time of extubation (T4), and 5 min after extubation (T5), the numerical rating scale (NRS) (0–10 points, no pain (score = 0) to unbearable pain (score = 10)), and at 0.5 h (T6), 4 h (T7), 8 h (T8), and 24 h (T9) after extubation. The secondary outcomes included the QoR-40 score on postoperative days 1 (POD1) and 2 (POD2), the consumption of sufentanil, propofol, and remifentanil during surgery, the incidence of postoperative nausea and vomiting, respiratory depression, urinary retention, pruritus, dizziness, and the need for rescue analgesia. In addition, the operation time, recovery time, and extubation time were recorded. The QoR-40 score is divided into five dimensions, including physical comfort (12 items), emotional state (9 items), physical independence (5 items), psychological support (7 items), and pain (7 items), with a total of 40 items. Each item is scored at 5 levels, with a minimum total score of 40 points and a maximum score of 200 points. The higher the score, the better the quality of recovery [7, 8]. In this study, the QoR-40 questionnaire was conducted on preoperative day 1 (Pre), postoperative day 1 (POD1), and postoperative day 2 (POD2).

### 2.3. Statistical Analysis

The main outcome was the postoperative NRS score. According to the relevant literature and pretest results, using PASS 11.0 software with a statistical power of 0.90 and an alpha level of 0.05, it was estimated that 30 patients in each group and 90 patients in total would be needed. Taking into account 5% losses, it was necessary to reach 32 patients in each group, so the needed sample size was 96 patients in total.

Statistical Package for Social Sciences (SPSS/version 20, IBM, Armonk, NY) software was used for statistical analysis. The measurement data of normal distribution were presented as mean ± SD and compared by one-way analysis of variance (ANOVA) between groups. Repeated measurement analysis of variance was used for comparison at different time points with Bonferroni correction. The measurement data and ranked data of abnormal distribution were expressed by median (*m*) and interquartile range (IQR), and the rank sum test was used for intergroup comparison; the count data were expressed in constituent ratio or rate (%) and compared between groups by the chi-square test or Fisher's exact test. *P* < 0.05 was statistically significant.

## 3. Results

### 3.1. Demographic Data

**Figure 1:**
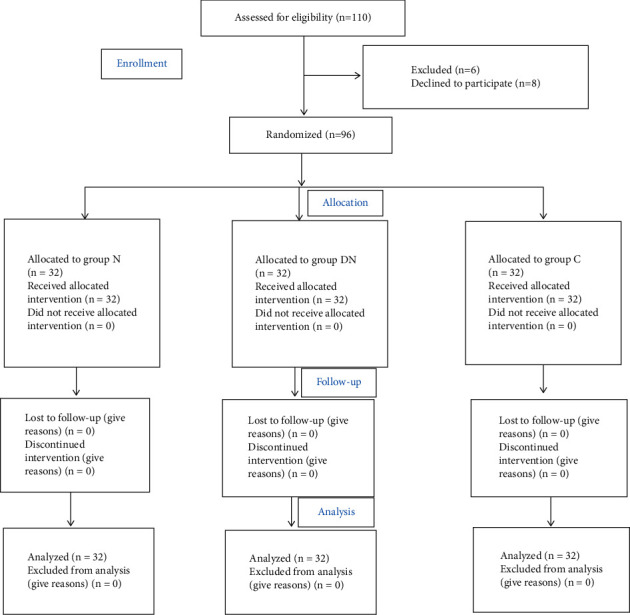
Flowchart showing details of clinical procedures throughout the study.

Initially, 110 patients were selected, of which 6 patients were excluded, 8 patients declined to complete the postoperative NRS scores, and the final sample size was 96 patients (Figure 1). There were no significant differences in terms of baseline data (*P* > 0.05, Table 1).

### 3.2. MAP at Different Time Points

Intragroup comparison: compared with T1, the mean arterial pressure in groups C and N at T2–T5 was significantly higher (*P* < 0.05), while there was no significant difference in group DN at T2–T5 (*P* > 0.05). Comparisons between groups: there was no significant difference in MAP among the three groups at T1 (*P*=0.585). Compared with group C, MAP in groups N and DN was significantly lower at T2–T5 (*P* < 0.001). Compared with group N, MAP in group DN at T2–T5 was comparatively lower (*P* < 0.001, Table 2).

### 3.3. HR at Different Time Points

Intragroup comparison: compared with T1, HR in groups C and N was significantly higher at T2–T5 (*P* < 0.05), but there was no significant difference in group DN at T2–T5 (*P* > 0.05). Comparisons between groups: there were no significant differences among the three groups at T1 (*P*=0.935). Compared with group C, HR in groups N and DN was significantly lower at T2–T5 (*P* < 0.001). Compared with group N, HR in group DN was comparatively lower at T2–T5 (*P* < 0.001, Table 2).

### 3.4. NRS Pain Scores

Compared with group C, NRS scores in groups N and DN were significantly lower at T6–T9 (*P* < 0.001). Compared with N, NRS scores in group DN were significantly lower at T6–T9 (*P* < 0.001, Table 3).

### 3.5. QoR-40 Scores

There were no significant differences in preoperative QoR-40 scores among the three groups (*P*=0.279), and the postoperative scores were significantly lower than those before surgery (*P* < 0.001). Compared with group C, QoR-40 scores in groups N and DN were significantly higher on POD1 and POD2 (*P* < 0.001). Compared with group N, QoR-40 scores in the DN group were higher on POD1 and POD2 (*P* < 0.001, Table 3).

### 3.6. The Consumption of Intraoperative Variables in the Three Groups

There were no significant differences in the dosage of sufentanil among the three groups (*P*=0.706). Compared with group C, the dosages of remifentanil and propofol in groups N and DN were significantly reduced (*P* < 0.0011). Compared with group N, the consumption of remifentanil and propofol in group DN was significantly reduced (*P* < 0.001). There were no significant differences in the awaking time and extubation time among the three groups (*P*=0.423; *P*=0.782, respectively, Table 4).

### 3.7. The Need for Rescue Analgesia and Side Effects

Compared with group C, the incidence of nausea and vomiting was lower in groups N and DN (*P*=0.003; *P* < 0.001, respectively), and there were no significant differences in pruritus, respiratory depression, urinary retention, or dizziness (*P* > 0.05). Compared with group C, the need for rescue analgesia in groups N and DN was decreased (*P*=0.008; *P* < 0.001, respectively); compared with group N, the need for rescue analgesia in group DN was decreased (*P*=0.012, Table 4).

## 4. Discussion

Preventive analgesia may help to reduce the incidence and severity of acute and chronic postoperative pain [9]. It may produce the analgesic effect beyond its intended duration due to reduced central sensitization. Nalbuphine is suitable for moderate to severe pain. It exerts sedative and analgesic effects by activating the kappa receptor. In addition, it antagonizes the respiratory depression of other opioids, increases the analgesic effect of these drugs, and treats opioid-induced pruritus as an antagonist at the mu-opioid receptor [10]. Compared with other opioids, nalbuphine has a short action time and rapid clearance and is less likely to cause side effects such as nausea and vomiting, itching, urinary retention, respiratory depression, and excessive sedation [11]. Dexmedetomidine is known for its analgesic potential due to its reduced sympathetic tone [12]. It exerts sedative and analgesic effects by binding to *α*2-adrenergic receptor agonists in the locus coeruleus and dorsal horn of the spinal cord [13]. It has been found that the combination of nalbuphine and dexmedetomidine for middle ear surgeries can maintain good sedation, provide an adequate analgesic effect during and after the surgery, and improve the satisfaction of patients and surgeons with a bloodless surgical field [14]. Kamal et al. found that the combination of nalbuphine and dexmedetomidine could improve analgesic and sedative effects and reduce the consumption of anesthetic [15]. This study aimed to observe the effect of nalbuphine combined with dexmedetomidine on hemodynamics and postoperative pain in patients undergoing endoscopic sinus surgery.

The results showed that the NRS score increased first and then decreased after endoscopic sinus surgery, and the pain peak was 4–8 h after surgery. The NRS scores at 0.5 h, 4 h, 8 h, and 24 h after extubation and the cases of rescue analgesia on POD1 and POD2 in the nalbuphine combined with dexmedetomidine group were the lowest, and the QoR-40 scores were the highest (*P* < 0.001), which indicated that the combination of nalbuphine and dexmedetomidine can improve the analgesic effect and the quality of postoperative recovery. The possible reasons for postoperative pain after endoscopic sinus surgery are as follows: surgical trauma causes the release of inflammatory factors and pain mediums and increases the synthesis of prostaglandins, which finally results in pain sensitization [16]; long-term sleep disorders caused by nasal diseases and the consumption of remifentanil during general anesthesia may lead to postoperative hyperalgesia [17]; and the effect of postoperative nasal packing on respiration will increase the incidence of headache and discomfort caused by brain hypoxia [18]. Nalbuphine inhibits the release of substance P from primary afferents by activating the kappa receptor in the spinal cord, thus reducing the transmission of the nerve impulses of pain to the central nervous system [19]. The activation of the kappa receptor induces an anti-inflammatory response through the downregulation of cytokine, chemokine, and chemokine receptor expression, and its anti-inflammatory effect is stronger than that of other receptors [20]. KOR agonists not only have analgesic activity but also exhibit anti-inflammatory activity and antinociceptive effect [21]. Nalbuphine could reduce the impact of surgical trauma on plasma through its anti-inflammatory and antioxidant effects [22]. Gong et al. found that nalbuphine could decrease the levels of inflammatory cytokines (IL-6, TNF-*α*, IL-1, and hs-CRP) in patients after fracture surgery [23]. Dexmedetomidine produces the antinociceptive effect by reducing NMDA receptor-mediated synaptic transmission. Chen et al. [24] showed that dexmedetomidine significantly decreased the levels of IL-6 and TNF-*α*, which demonstrated its anti-inflammatory effect. It is well known that the stimulation of *α*2 adrenoreceptors in the spinal cord plays an important role in analgesia. Studies found that the combination of nalbuphine and dexmedetomidine showed better analgesic and sedative effects, and the level of inflammatory factors decreased [25], and their results were consistent with ours. The mechanism that the analgesic effect of nalbuphine combined with dexmedetomidine is better may be that they have a synergistic antinociceptive effect by activating *α* 2 adrenoreceptors and kappa receptors, and dexmedetomidine can enhance the analgesic effect of opioids and reduce the demand for opioid and nonopioid analgesics [26, 27].

Compared with T1, MAP and HR of groups N and C were significantly increased in T2–T5 (*P* < 0.05), but there was no significant difference in the DN group (*P* > 0.05). In addition, MAP and HR in the DN group were the lowest (*P* < 0.001), indicating that nalbuphine combined with dexmedetomidine could maintain the stability of hemodynamics, and the effect was the best among the three groups. Nalbuphine is used to attenuate the hemodynamic response to laryngoscopy and endotracheal intubation because of its longer analgesic duration and almost no respiratory depression and cardiovascular side effects [28–30]. Chawda et al. [31] found that 0.2 mg/kg nalbuphine given 3–5 minutes before intubation can effectively prevent the pressor response. Dexmedetomidine, with sedative, analgesic, and antisympathetic effects, can inhibit the release of catecholamine by activating the *α*2 receptor and attenuate the stress response to surgery, thereby maintaining stable hemodynamics [32, 33]. The reason why the effect of nalbuphine combined with dexmedetomidine is better may be related to the additive or synergistic effect of the two drugs.

This study found that the consumption of remifentanil and propofol during the operation and the incidence of postoperative nausea and vomiting in the combined group were the lowest (*P* < 0.001), indicating that the combination of nalbuphine and dexmedetomidine for preventive analgesia could reduce the usage of anesthetics during the operation and the incidence of postoperative adverse reactions, and the effect was much better than that of the other two groups. Some studies have found that nalbuphine can not only relieve pain but also reduce the dosage of propofol and the incidence of adverse reactions during surgery [34], which is consistent with the results of this study. Studies have discovered that perioperative application of dexmedetomidine can reduce opioid consumption and decrease the degree of pain and the incidence of nausea. The better effect of the combination group may be related to the additive or synergistic effect of the two drugs. In some studies, dexmedetomidine in combination with anesthetics, sedatives, or opioids may enhance sedative and analgesic effects [5].

There are some limitations to the study. First, this study was a small sample and needed to be verified by more high-quality, multicenter randomized controlled trials. Second, this study did not compare the different doses of the two drugs. The optimal dose of the two drugs in combination needs to be further studied.

## 5. Conclusion

In general, preventive analgesia with nalbuphine and dexmedetomidine in endoscopic sinus surgery can not only maintain hemodynamic stability but also reduce the intraoperative anesthetic dosage, the degree of postoperative pain, and the incidence of adverse reactions and improve the quality of postoperative recovery.

## Figures and Tables

**Table 1:** Demographics of the participants who completed the study.

Item	Group N	Group DN	Group C	*P* value
Age (years)	39.69 ± 2.21	40.34 ± 1.92	37.03 ± 1.82	0.463
BMI (kg/m^2^)	23.29 ± 0.46	23.08 ± 0.39	22.85 ± 0.42	0.774
Sex (male/female)	19/13	13/19	14/18	0.274
ASA I/II	17/15	14/18	17/15	0.687
Operation time (min)	53.97 ± 2.19	57.84 ± 2.14	52.06 ± 1.70	0.125

Values are expressed as mean ± SD or number of patients. ASA, American Society of Anesthesiologists; BMI, body mass index; group N, preoperative nalbuphine injection; group DN, preoperative nalbuphine combined with dexmedetomidine injection; group C, preoperative saline injection.

**Table 2:** Hemodynamic values.

Measurement time		Group N	Group DN	Group C
HR values	T1	72.84 ± 1.48	72.59 ± 1.48	73.34 ± 1.48
	T2	85.41 ± 0.80^a^^*∗*^	74.78 ± 0.80^ab^	93.47 ± 0.80^*∗*^
	T3	84.53 ± 0.69^a^^*∗*^	74.84 ± 0.69^ab^	91.63 ± 0.69^*∗*^
	T4	82.69 ± 0.79^a^^*∗*^	75.22 ± 0.79^ab^	91.16 ± 0.79^*∗*^
	T5	77.66 ± 1.01^a^^*∗*^	71.56 ± 1.01^ab^	81.28 ± 1.01^*∗*^

MAP values	T1	92.38 ± 1.01	93.22 ± 1.01	91.75 ± 1.01
	T2	102.56 ± 0.84^a^^*∗*^	94.66 ± 0.84^ab^	111.28 ± 0.84^*∗*^
	T3	103.47 ± 0.89^a^^*∗*^	94.75 ± 0.89^ab^	111.97 ± 0.89^*∗*^
	T4	102.78 ± 0.91^a^^*∗*^	94.81 ± 0.91^ab^	116.34 ± 0.91^*∗*^
	T5	97.72 ± 1.02^a^^*∗*^	94.19 ± 1.02^ab^	102.75 ± 1.02^*∗*^

Values are presented as mean ± SD. HR, heart rate; MAP, mean arterial pressure; group N, preoperative nalbuphine injection; group DN, preoperative nalbuphine combined with dexmedetomidine injection; group C, preoperative saline injection. ^*∗*^*P* < 0.05 compared with T1 in the same group; ^a^*P* < 0.05 compared with group C; b*P* < 0.05 compared with group N.

**Table 3:** Comparison of the NRS scores and QoR-40 scores between the three groups.

Variable		Group N	Group DN	Group C
NRS scores	T6	0.97 ± 0.08^a^	0.06 ± 0.08^ab^	3.03 ± 0.08
	T7	3.50 ± 0.14^a^	2.16 ± 0.14^ab^	5.72 ± 0.14
	T8	2.78 ± 0.14^a^	1.78 ± 0.14^ab^	5.00 ± 0.14
	T9	2.16 ± 0.11^a^	1.00 ± 0.11^ab^	4.09 ± 0.11

QoR-40 scores	Pre	195.09 ± 0.27	195.19 ± 0.27	195.66 ± 0.27
	POD1	178.88 ± 1.13^a^	186.2 ± 1.13^ab^	173.03 ± 1.13
	POD2	183.81 ± 0.95^a^	193.3 ± 0.95^ab^	177.78 ± 0.95

Values are presented as mean ± SD. NRS, numeric rating scale; QoR-40, quality of recovery-40; POD, postoperative day; group N, preoperative nalbuphine injection; group DN, preoperative nalbuphine combined with dexmedetomidine injection; group C, preoperative saline injection. ^a^*P* < 0.05 compared with group C; ^b^*P* < 0.05 compared with group N.

**Table 4:** Comparison of intraoperative variables, side effects, and the need for rescue analgesia in the three groups.

	Group N	Group DN	Group C	*P* value
Intraoperative variables				
Awaking time (min)	21.16 ± 0.75	20.44 ± 0.73	19.81 ± 0.70	0.423
Extubation time (min)	23.00 ± 0.73	22.34 ± 0.73	22.38 ± 0.78	0.782
Remifentanil dosage (ug)	249.69 ± 5.93^a^	176.88 ± 7.53^ab^	296.56 ± 8.85	<0.001
Sufentanil dosage (ug)	34.38 ± 0.66	33.75 ± 0.71	34.53 ± 0.72	0.706
Propofol dosage (mg)	340.94 ± 7.92^a^	262.81 ± 6.46^ab^	397.50 ± 8.39	<0.001

Side effects				
Nausea and vomiting	2 (6.3%)^a^	0^a^	12 (37.5%)	<0.001
Pruritus	0	0	2 (6.3%)	0.130
Respiratory depression	0	0	2 (6.3%)	0.130
Urinary retention	2 (6.3%)	1 (3.1%)	3 (9.4%)	0.587
Dizziness	0	2 (6.3%)	3 (9.4%)	0.228
The need for rescue analgesia	6 (18.8%)^a^	0^ab^	16 (50%)	<0.001

Group N, preoperative nalbuphine injection; group DN, preoperative nalbuphine combined with dexmedetomidine injection; group C, preoperative saline injection. ^a^*P* < 0.05 compared with group C; ^b^*P* < 0.05 compared with group N.

## Data Availability

No data were used to support the findings of this study.

## References

[1] Guntinas-Lichius O., Volk G. F., Zaslansky R., Meissner W. (2014). The first postoperative day. *The Clinical Journal of Pain*.

[2] Long J. B., Bevil K., Giles D. L. (2019). Preemptive analgesia in minimally invasive gynecologic surgery. *Journal of Minimally Invasive Gynecology*.

[3] Durmus M., But A. K., Dogan Z., Yucel A., Miman M. C., Ersoy M. O. (2007). Effect of dexmedetomidine on bleeding during tympanoplasty or septorhinoplasty. *European Journal of Anaesthesiology*.

[4] Blaudszun G., Lysakowski C., Elia N., Tramèr M. R. (2012). Effect of perioperative systemic *α*2 agonists on postoperative morphine consumption and pain intensity. *Anesthesiology*.

[5] Kaye A. D., Chernobylsky D. J., Thakur P. (2020). Dexmedetomidine in enhanced recovery after surgery (ERAS) protocols for postoperative pain. *Current Pain and Headache Reports*.

[6] Davis M. P., Fernandez C., Regel S., McPherson M. L. (2018). Does nalbuphine have a niche in managing pain?. *Journal of Opioid Management*.

[7] Lee J. H., Kim D., Seo D., Son J.-s., Kim D.-C. (2018). Validity and reliability of the Korean version of the quality of recovery-40 questionnaire. *Korean Journal of Anesthesiology*.

[8] Terkawi A., Myles P., Riad W. (2017). Development and validation of Arabic version of the postoperative quality of recovery-40 questionnaire. *Saudi Journal of Anaesthesia*.

[9] Luo J., Min S. (2017). Postoperative pain management in the postanesthesia care unit: an update. *Journal of Pain Research*.

[10] Portugal F., Mehta Z., Davis M. (2019). Nalbuphine^#^ 381. *Journal of Palliative Medicine*.

[11] Zeng Z., Lu J., Shu C. (2015). A comparision of nalbuphine with morphine for analgesic effects and safety: meta-analysis of randomized controlled trials. *Scientific Reports*.

[12] Barends C. R. M., Absalom A., van Minnen B., Vissink A., Visser A. (2017). Dexmedetomidine versus midazolam in procedural sedation a systematic review of efficacy and safety. *PLoS One*.

[13] Du X., Yu J., Mi W. (2018). The effect of dexmedetomidine on the perioperative hemodynamics and postoperative cognitive function of elderly patients with hypertension. *Medicine*.

[14] Nallam S., Chiruvella S., Reddy A. (2017). Monitored anaesthesia care—comparison of nalbuphine/dexmedetomidine versus nalbuphine/propofol for middle ear surgeries: a double-blind randomised trial. *Indian Journal of Anaesthesia*.

[15] Kamal N. M., Radwan T. A., Mohamed A. A. (2019). Dexmedetomidine as an adjuvant to nalbuphine in patient controlled analgesia for post-operative pain in laparoscopic cholecystectomy: a preliminary study. *Global Journal of Anesthesiology*.

[16] Li L., Wu Y., Bai Z., Hu Y., Li W. (2017). Blockade of NMDA receptors decreased spinal microglia activation in bee venom induced acute inflammatory pain in rats. *Neurological Research*.

[17] Yu E. H. Y., Tran D. H. D., Lam S. W., Irwin M. G. (2016). Remifentanil tolerance and hyperalgesia: short-term gain, long-term pain?. *Anaesthesia*.

[18] Huang G. H., Hao Y. Y., Wang T. L., Chen B. S., Wang G. F. (2018). Assessment of perioperative arterial oxygen and carbon dioxide pressure by nasal packing versus transseptal suturing techniques after septoplasty. *China Journal of Endoscopy*.

[19] Inan S., Torres-Huerta A., Jensen L. E., Dun N. J., Cowan A. (2019). Nalbuphine, a kappa opioid receptor agonist and mu opioid receptor antagonist attenuates pruritus, decreases IL-31, and increases IL-10 in mice with contact dermatitis. *European Journal of Pharmacology*.

[20] Finley M. J., Happel C. M., Kaminsky D. E., Rogers T. J. (2008). Opioid and nociceptin receptors regulate cytokine and cytokine receptor expression. *Cellular Immunology*.

[21] Aldrich J. V., McLaughlin J. P. (2009). Peptide kappa opioid receptor ligands: potential for drug development. *The AAPS Journal*.

[22] Xi M. Y., Li S. S., Zhang C., Zhang L., Wang T., Yu C. (2020). Nalbuphine for analgesia after orthognathic surgery and its effect on postoperative inflammatory and oxidative stress: a randomized double-blind controlled trial. *Journal of Oral and Maxillofacial Surgery*.

[23] Gong Y., Zhang Y., Tao S. (2018). Nalbuphine for analgesia after fracture surgery and its effect on circulating inflammatory factors. *Experimental and Therapeutic Medicine*.

[24] Chen W., Liu B., Zhang F., Xue P., Cui R., Lei W. (2015). The effects of dexmedetomidine on post-operative cognitive dysfunction and inflammatory factors in senile patients. *International Journal of Clinical and Experimental Medicine*.

[25] Liu X., Song J., Zhang Y., Zhang Y., Hu X. (2021). Different doses of nalbuphine combined with dexmedetomidine in laparoscopic oophorocystectomy. *Medical Science Monitor*.

[26] Song Y., Shim J.-K., Song J.-W., Kim E.-K., Kwak Y.-L. (2016). Dexmedetomidine added to an opioid-based analgesic regimen for the prevention of postoperative nausea and vomiting in highly susceptible patients. *European Journal of Anaesthesiology*.

[27] Unal M., Gursoy S., Altun A. (2013). Ineffective doses of dexmedetomidine potentiates the antinociception induced by morphine and fentanyl in acute pain model. *Korean Journal of Physiology and Pharmacology*.

[28] Fragen R. J., Caldwell N. (1977). Acute intravenous premedication with nalbuphine. *Anesthesia and Analgesia*.

[29] Klepper I. D., Rosen M., Vickers M. D., Mapleson W. W. (1986). Respiratory function following nalbuphine and morphine in anaesthetized man. *British Journal of Anaesthesia*.

[30] Lake C. L., Duckworth E. N., DiFazio C. A., Magruder M. R. (1984). Cardiorespiratory effects of nalbuphine and morphine premedication in adult cardiac surgical patients. *Acta Anaesthesiologica Scandinavica*.

[31] Chawda P., Pareek M., Mehta K. (2010). Effect of nalbuphine on haemodynamic response to orotracheal intubation. *Journal of Anaesthesiology Clinical Pharmacology*.

[32] Gupta K., Bansal M., Gupta P., Pandey M., Agarwal S. (2015). Dexmedetomidine infusion during middle ear surgery under general anaesthesia to provide oligaemic surgical field: a prospective study. *Indian Journal of Anaesthesia*.

[33] Sudheesh K., Harsoor S. (2011). Dexmedetomidine in anaesthesia practice: a wonder drug?. *Indian Journal of Anaesthesia*.

[34] Li H. Y., Li H. F., Ren Y. F., Zheng X. Z., Mao S. S., Pang H. L. (2020). Application of nalbuphine combined with propofol in painless endoscopic retrograde cholangiopancreatography in patients over age 60. *International Journal of Anesthesiology and Resuscitation*.

